# Multifaceted modeling of small intestinal neuroendocrine tumors

**DOI:** 10.1530/EO-24-0038

**Published:** 2024-11-20

**Authors:** Stephen Gabriel Andrews, Steven D Forsythe, James P Madigan, Samira Mercedes Sadowski

**Affiliations:** 1 Neuroendocrine Cancer Therapy Section, Surgical Oncology Program, Center for Cancer Research, National Cancer Institute, National Institutes of Health, Bethesda, Maryland, USA

**Keywords:** small intestinal neuroendocrine tumors, organoid, cell lines, animal models, radiomics, therapy

## Abstract

Small intestinal neuroendocrine tumors, siNETs, are a group of rare cancers that arise from neuroendocrine cells in the lining of the jejunum and ileum, which are either classified as tumors, siNETs, or small intestinal neuroendocrine carcinomas, siNECs. Current treatment strategies for low-grade tumors include surgical resection, peptide radionucleotide receptor therapy, and somatostatin analogues, while high-grade and recurrent tumors may receive cytotoxic chemotherapy. These limited treatment options are linked to the lack of representative models that can both reflect the biology of the tumor and are amenable to mid-to-high throughput experimentation. Cell line generation is challenging considering the indolent nature of primary lesions, although some attempts have been successful using a variety of methods and include the primary P-STS line and those derived from metastatic lesions, including GOT1, CNDT2.5, and HC45. Patient-derived modeling, including organoids and xenografting, have allowed for multicellular and 3D representations of the original tumor. These specific models allow for multicellular populations derived from the tumor, providing better tumor representation for use in drug screening and *in vitro* assays. Currently, there are limited, although increasing, published models of siNETs implanted as xenografts in mice and zebrafish. As these cellular and animal models provide insights into siNET biology, theragnostic modeling has provided key information on the clinical progression and treatment of this disease. Significant strides toward more representative models have been made throughout the last decade. In this review, details of these attempts as well as future directions and strategies for more robust models will be addressed.

## Introduction

Small intestinal neuroendocrine tumors (siNETs) are a rare malignancy of the midgut, located within the jejunum and ileum between the Ligament of Treitz and the ileocecal valve (Modlin *et al.* 2004). Although uncommon overall, these slow-growing tumors are the most frequent malignancy in the small intestine and are increasing in incidence and prevalence in the United States, with an annual incidence of about 1.05 per 100,000 (Dasari *et al.* 2017). There has been a rise in the incidence of this pathology, which can be attributed to better diagnostic strategies, imaging, and clinical awareness (Dasari *et al.* 2017). Recently, the incidence of siNETs has been reported at 1.46 per 100,000 (White *et al.* 2022). In some cases, these tumors can contribute to carcinoid syndrome (Kerstrom *et al.* 2005). Carcinoid syndrome is a paraneoplastic disease caused by the release of hormones, primarily serotonin, which results in flushing, diarrhea, and, rarely, carcinoid heart disease. In the past, these tumors were generally were classified as midgut carcinoids; however, the nomenclature of these malignancies has been reclassified to reflect molecular and cellular differences within this group as siNETs and small intestinal neuroendocrine carcinomas (siNECs). Modeling is vital for the improvement of imaging techniques, diagnosis, targeted therapeutics, and patient outcomes for siNETs of all grades and metastatic statuses. There have been some analyses of broad gastro-entero-pancreatic neuroendocrine tumor models published previously (Kawasaki *et al.* 2018); however, this review aims to provide an in-depth portrayal of small intestinal NET models on a molecular, cellular, and organismal level. Notably, functional small cell and ampullary duodenal NETs will not be included in this review, as they are genetically and clinically separate from other siNETs.

SiNETs are inherently indolent in comparison to many other cancers, including cancers in the neuroendocrine category which originate in other tissues. SiNETs have a low mutational burden, with *CDKN1B* locus alteration, the most commonly observed mutation, present in fewer than 10% of cases (Francis *et al.* 2013). However, hemizygous loss of chromosome 18q, which contains the *SMAD4* gene, has been reported to be present in 74% of patients in one study (Hofving *et al.* 2021*a*). This relatively low mutation burden complicates the creation of models, which often depend on multiple genetic abnormalities to improve establishment; alternatively, increased knowledge of the somatic copy number alterations that underlie this cancer may provide insights for improved model creation. Models that represent the biology of SiNETs are essential for preclinical and translational studies that improve patient outcomes. In addition, given the relative scarcity of fresh tumor samples, models are necessary to recapitulate this neoplasm with sufficient breadth and depth to improve the diagnosis, treatment, and prognosis of patients with this disease. Overall, the best models to study siNETs are those that reflect the genetic alterations, histological markers, and behavior of most tumors in this category.

**Figure 1:**
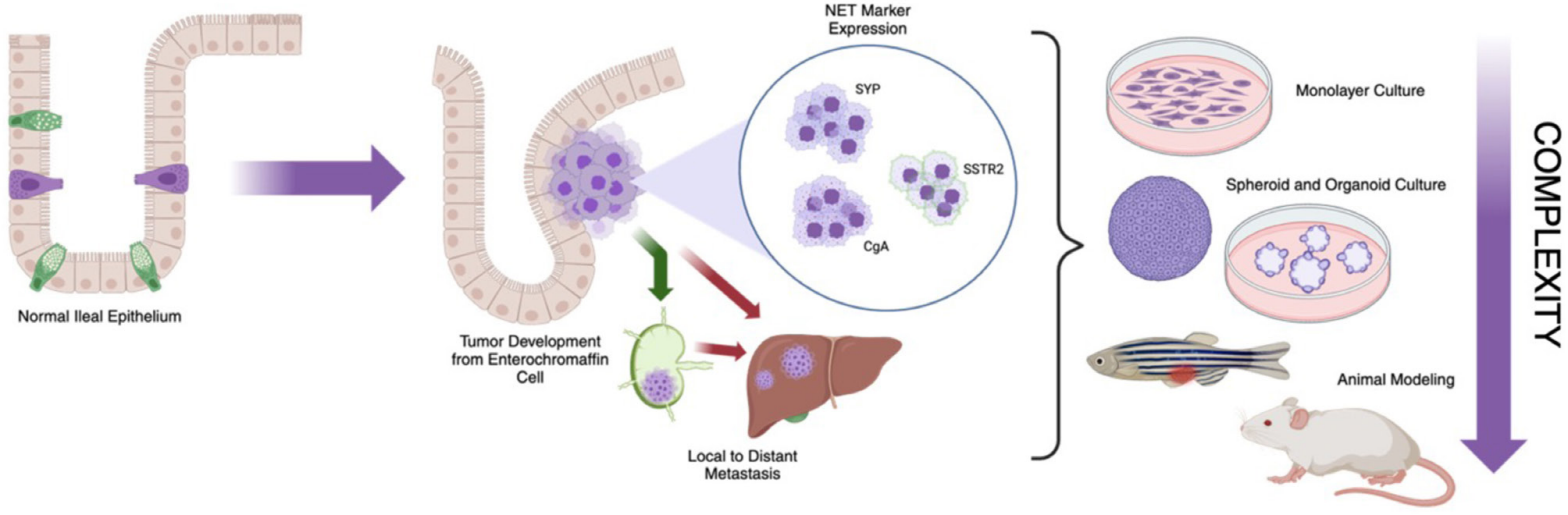
Schematic describing the proposed development and progression of small intestinal neuroendocrine tumors, as well as current models at an increasing level of complexity and disease representation. Image created with Biorender.com.

Current treatments for siNETs are similar to NETs of other origins, including the pancreas, colon, and rectum (Pavel *et al.* 2020). SiNETs are graded in the same fashion as other malignancies, via the WHO system; grade 1 (G1) corresponds to a Ki-67 of less than 3%, grade 2 (G2) to a Ki-67 of 3–20%, and grade 3 (G3) above 20%. In G1 and G2, primary lesions are surgically resected, with a lower risk of recurrence. Well-differentiated tumors are also candidates for PRRT and somatostatin analogue therapy, which targets SSTR2-expressing tumor cells. In higher grades, Ki-67 index >20%, cytotoxic chemotherapy is the primary symptom-attenuation treatment option. Therefore, there is a strong need for more effective and varied treatment strategies to improve outcomes and the quality of life for siNET patients, especially those with G3 siNETs and siNECs. Powerful modeling is a requirement for such advancement in therapy. From least to most complex, these models include cell lines, spheroids, organoids, and animal models; however, as complexity and tumor representation increase, researchers are limited by throughput (Fig. 1). The overall aim of this review is to provide a detailed discussion of the utilization and limitations of siNET models and address areas of future research in this field.

## Cell lines

Monolayer culture of immortalized cells derived from tumor samples is one of the oldest and most utilized techniques to model cancers. They can be used in a high-throughput format that is amenable to large-scale genetic interrogation, drug screening, and various molecular assays. However, due to the slow-growing nature of low-grade siNETs, deriving cell lines from primary tissue has been challenging. Most lesions will be positive for Chromogranin A, Synaptophysin, and Neuron-specific Enolase (NSE), among others, but there are examples of siNETs that do not express one or a combination of these biomarkers (Table 1). To date, there has only been one published cell line derived from a non-functional human primary lesion and three cell lines derived from liver metastases (Table 2). These lines vary in doubling time, immunohistochemical profile, and original patient pathology, which reflect the heterogeneity of this pathology broadly.

**Table 1:** Immunohistological markers of siNETs and siNET model development.

Marker	Function	Relevance to siNETs and siNET models	Cellular location	References
*Synaptophysin (Syp)*	Integral membrane protein in neural synaptic vesicles	Expressed by almost all siNETs with high senstitivity	Tumor cell intracytoplasmic vesicles	Miettinen (1987), Andersson *et al.* (2016)
*Chromogranin A (CgA)*	Acidic secretory pro-hormone protein from neurons and most neuroendocrine cells	Main biomarker for the clinical diagnosis of siNETs, with high specificity and medium-to-high sensitivity	Cytoplasmic secretory granules	D’Amico *et al.* (2014), Andersson *et al.* (2016), Di Giacinto *et al.* (2018)
*Somatostatin receptor 2 (SSTR2)*	Membrane receptor for the cyclic peptide, growth-hormone-inhibiting hormone somatostatin	Found in a majority of siNETs, functional target for siNET therapy	Within the cytoplasm and within the plasma membrane	Papotti *et al.* (2002), Watanabe *et al.* (2022)
*Neuron-specific enolase (NSE)*	Isoenzyme of the glycolytic enzyme enolase, secreted in late-stage neural maturation	Upregulated in tumors and analyzed in coordination with Syp and CgA	Dispersed throughout the cytoplasm, occasionally in the nucleus and on the cell surface	Isgro *et al.* (2015), Mjones *et al.* (2017)
*Vesicular monoamine transporter 1 (VMAT1)*	Mediate amine transport into vesicles	Secreted by amine-producing neuroendocrine tumors of ileal origin	Found in large dense core and small synaptic vesicles	Erickson *et al.* (1996), Jakobsen *et al.* (2001)
*Serotonin (5-HT)*	Vasoactive hormone secreted by many endocrine organs, including enterochromaffin cells	Marker used in the diagnosis of siNET, present in >85% of tumors	Diffuse throughout the cytoplasm	Yang *et al.* (1983), Krishnamurthy & Dayal (1997), Blazevic *et al.* (2018)
*Substance P*	Neuropeptide that is widely present in neural cells and promotes tumor cell migration	Positive in some tumors and cell lines	Cytoplasmic vesicles	Hofving *et al.* (2018), Covenas & Munoz (2022)

**Table 2:** Known siNET cell lines, their origin, immunohistochemical profile, growth profile, and original publication.

Cell line (original publication)	Origin	Immunohistochemical profile	Growth profile	References
*P-STS*	G3 tumor of the terminal ileum	Pan-CK, serotonin, CD56, Syp, HISL19, NSE	Doubling time: 4 days–1 week, grows in mildly adherent culture with some non-adherent aggregates	Pfragner *et al.* (2009), Hofving *et al.* (2018)
*GOT-1*	G1 carcinoid tumor liver metastasis	CgA, 5-HT, VMAT1, serotonin, NCAM	Doubling time: 18 days	Kölby *et al.* (2001), Hofving *et al.* (2018)
*CNDT2.5*	G1 liver metastasis from an ileal carcinoid	Syp, NSE, produced serotonin, SSTR1-5	Limited published data	Van Buren *et al.* (2007), Barazeghi *et al.* (2021)
*HC45*	G1 liver metastasis from an ileal carcinoid	CgA, CgB, SSTR2, SSTR5, Syp, VMAT 1, vitamin D3 receptor	Limited published data	Stilling *et al.* (2007)
*SS-C*	Jejunal somatostatinoma	SSTR2, NSE, CgA, Syp	Doubling time: 3–6 days	Galli *et al.* (2006)
*STC-1 (mouse)*	Tumor from the duodenum of a RIP1Tag2/RIP2PyST1 mouse	Secretin, gastrin, somatostanin, CgA	Doubling time: 54 h	Rindi *et al.* (1990)

### Non-functional primary siNET cell line

#### P-STS

In 2009, Pfragner *et al.* (2009) reported the development of a stable cell line derived from a siNET, specifically a primary lesion from the distal ileum of a 42-year-old woman that originated from enterochromaffin cells. P-STS cells form loose monolayers with an average doubling time of approximately 4 days. Upon initial culture, P-STS expressed pancytokeratin, cytokeratins 7, 8, 18, and 19, serotonin, NSE, CD56, PGP9.5, calcitonin, synaptophysin, and growth hormone-releasing factor. This further establishes this line as a representative model of the original lesion and its neuroendocrine phenotype (Pfragner *et al.* 2009). Although most cells were adherent in culture, floating cells displayed chromosomal alterations at the 18q site, which is consistent with some studies showing losses and gains of 18q play a role in tumorigenesis and tumor progression in siNETs (Kytola *et al.* 2001). Of interest, genetic sequencing of the P-STS line showed no alterations to the *menin* coding region, suggesting sporadic tumor generation without the genetic abnormalities associated with MEN1 syndrome.* Hofving et al.* reported that the P-STS line displayed a neuroendocrine phenotype, and were responsive to HDAC inhibitors (Hofving *et al.* 2018). Recently, the P-STS cell line was utilized to investigate stromal and neoplastic cell crosstalk. The authors found that integrin signaling pathways play a role in tumor and stromal cell crosstalk that contributes to tissue fibrosis (Laskaratos *et al.* 2021). The original patient presented with local lymph node and hepatic metastases, which resulted in the development of L-STS and H-STS (Pfragner *et al.* 2009), respectively; however, these lines have been shown to be lymphoblastoid and not representative of siNETs.

### Metastatic siNET cell lines

#### GOT-1

In 2001*,* Kölby *et al.* reported the establishment of GOT-1 cells from a hepatic metastasis of a distal ileal NET of a 55-year-old woman, who also presented with lymph node metastases. The original tumor cells were positive for 5-HT, substance P, VMAT1/2, and markers for neuroendocrine phenotype, namely CgA, SYP, SV2, and NCAM, via IHC; the original tumor and the derived cell line also expressed somatostatin receptors, including SSTR2, which are the main treatment targets for these malignancies. Cell culture showed similar positive IHC results; therefore, the identity of the original GOT-1 cell line was successfully verified. This cell line is still widely used for a variety of *in vitro* studies; however, the doubling time of more than 2 weeks (>18 days) limits its usage in proliferation and growth studies (Kölby *et al.* 2001, Grozinsky-Glasberg *et al.* 2012). This line also displays a loss of a 1.8 Mb segment of chromosome 18; this is representative of the original neoplastic patient sample and is aligned with chromosome loss that often accompanies siNETs (Hofving *et al.* 2018).

#### CNDT 2.5

Van Buren *et al.* (2007) reported the generation of the CNDT 2.5 cell line in 2007, derived from the primary CNDT2 monolayer culture, which originated from a liver metastasis of a primary ileal carcinoid. CNDT2 cells were grown and then injected into nude mice. Cells from the xenograft tumor were then isolated and cultured independently. The resultant CNDT 2.5 line was validated as siNET-derived via molecular, protein, and cellular assays. Grown in culture, these cells have an approximate doubling time of 20 h, allowing for efficacious growth and proliferation studies, *in vitro* drug screening, and other functional assays. CNDT 2.5 cells contain neurosecretory granules and express mRNA for NSE, SYP, and VEGF and its receptors. Additionally, these cells express many targetable tyrosine kinase receptors and all five SSTRs, which were proven to be functional; these cells also contain serotonin and serotonin receptors (Van Buren *et al.* 2007). Notably, the original culture of these cells did not express chromogranin A nor cytokeratins. This deviation led to some debate on the origin of the cell line (Ellis *et al.* 2010). Chromosomal aberrations were present in this model, but on different chromosomes and loci than in other siNET models, which commonly occur at chromosome 18. The authors reported deletions in 2p and 6q and various translocations. This data may suggest an alternate origin; however, there is no definitive evidence that the cell line is misclassified beyond the loss of some cellular markers. CNDT 2.5 is still used to some degree as a model for *in vitro* experiments. In 2019, one study investigated transmembrane receptor type tyrosine phosphatases in these cells. The researchers found that a specific protein tyrosine phosphatase, PTPRM, acted as a tumor suppressor in siNET cells (Barazeghi *et al.* 2019).

#### HC45

Stilling *et al.* (2007) published the initial culture of HC45, a transformant cell line from a liver metastasis of an ileal carcinoid, in 2007. An SV40 early T-antigen insert is a notable feature of this specific line, which alters the line’s genome to immortalize the cell line for future culture and propagation. The neuroendocrine cellular phenotype was verified via IHC; this line was positive for synaptophysin, chromogranin A/B, 7B2, SSTR2, and SSTR5, and rarely VMAT1. The cells were negative for serotonin. Uniquely, the HC45 expresses the vitamin D3 receptor. Western blotting of the original culture displayed high expression of TGFβR1 and TGFβR2, as well as EGFR, which proved capable of therapeutic targeting. This profile reflects the neuroendocrine phenotype of these cells and the hepatic metastasis from which this line was cultured. Often TGFβ expression is associated with cell proliferation and tumor metastasis in the later stages of disease (Xie *et al.* 2018). Additionally, EGFR plays a role in cell division and growth in many cancer types (Sasaki *et al.* 2013). This cell line has been used as an ileal NET comparison model in a study regarding viral oncogenesis in lung NETs (Haley *et al.* 2010) and has been cited as an appropriate model of liver metastasis of siNETs (Carpizo & Harris 2021).

### Functional and non-human siNET cell lines

#### SS-C

Galli *et al.* (2006) initially reported the generation of the SS-C cell line from a jejunal somatostatinoma that was surgically resected from a 28-year-old female. Somatostatinomas are incredibly rare among siNETs, making up less than 5% of cases. Notably, the authors mentioned that the original patient did not have any outlying hereditary or sporadic endocrine pathologies, including MEN1. Cultures of the primary tumor were positive for many neuroendocrine markers over several passages, including CgA, NSE, somatostatin, SSTRs, and gastrin, among others. These cells grow in a firm monolayer and have a doubling time of approximately 3–5 days, with an increase in days as passage number increases. This model represents a rare tumor that displays phenotype and functional differences from the large majority of siNETs.

#### STC-1 (mouse)

Rindi *et al.* (1990) reported the development of a murine cell line model of a small intestinal neuroendocrine adenoma, specifically from the duodenum, in 1990. This cell line was cultured from a RIP1Tag2/Rip2pyST1 transgenic mouse. The doubling time of this line is approximately 54 h. Upon immunohistochemical analysis (IHC), tumor cells stained positively for secretin, glicentin, gastrin, and somatostatin, albeit the latter was not ubiquitously expressed among the cells. Notably, only a select number of cells expressed both CgA and large T-antigen immunoreactivity; however, there was no mention of SYP. Although this is a murine-derived line, this calls the relative representation of the small intestinal neuroendocrine phenotype of STC-1 into question. Nevertheless, this line has been widely cited in both primary and review literature regarding both cancer and gastrointestinal metabolic disease. Most recently, STC-1 has been used experimentally as a model of cholecystokinin release in response to fermentation products (Egberts *et al.* 2020) and to test a cytotoxic herpes simplex virus on tumor growth in neuroendocrine cancer (Matsushima *et al.* 2019). Overall, this line may be useful in developing stable cell-derived xenograft tumor models in mice given inferred immuno-compatibility; however, the genetic and molecular differences between this model and human NETs must be considered.

## Organoid and spheroid modeling

**Figure 2:**
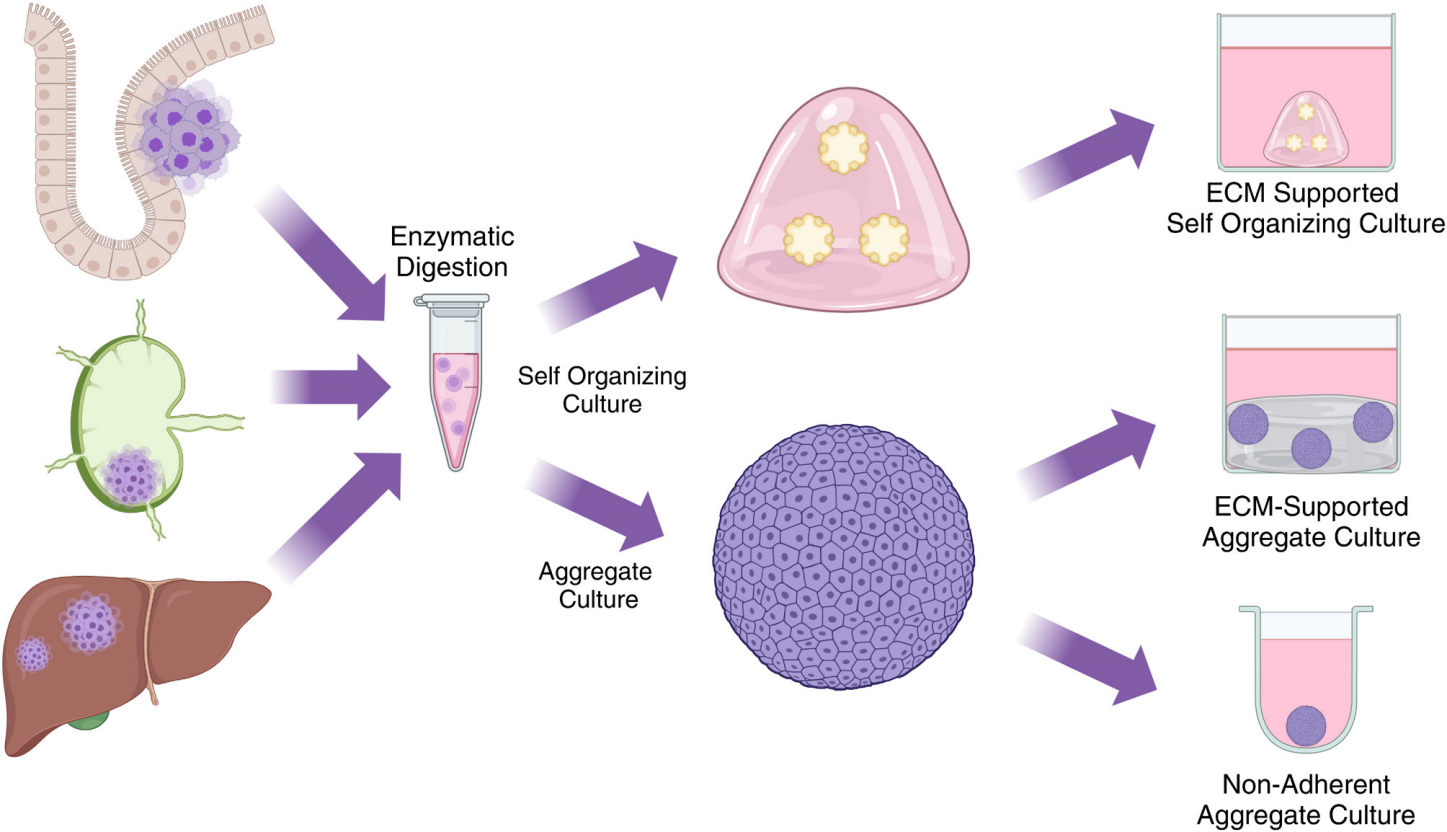
siNET 3D culture methodology. Image created with Biorender.com.

3D modeling presents a higher-order approach to studying siNETs and carcinomas *in vitro* (Fig. 2). This method provides a more representative view of how tumors behave, as opposed to a monolayer culture; yet they are still reproducible enough to perform high-throughput drug screening and cell-based assays. This section will primarily focus on patient-derived tumor organoids (PDTOs), which are 3D patient-derived cell structures present in a scaffold, often composed of extracellular matrix (ECM) components such as collagen or Matrigel. They can either self-organize into organ-like structures or form large aggregates of cells similar to tumor growth. These PDTOs can be cultured for multiple passages and stored for future experiments.

In comparison to siNET cell lines, some of which do not accurately reflect the genetic profile, patient-derived tumor cell organoids that are cultured *ex vivo* can provide more powerful and translational insights in target validation and therapy evaluation. Additionally, siNET 3D culture can take on molecular, cellular, and structural characteristics that mimic the original midgut tumor. This provides an avenue for target elucidation, drug screening, and mechanistic studies of small intestinal neoplastic cells that truly reflect the tumor biology and may lead to clinical applications to improve prognosis at a faster rate.

To date, there are only a few accounts of 3D cultures of siNET tumor cell populations developed from patient tissue derived from freshly resected siNET tumors. Ear *et al.* (2019) developed a strategy for developing these 3D structures that support long-term culture for more than 9 months continuously, allowing for drug screening and functional studies downstream. Although the authors of this publication refer to their cultures as spheroids, the use of a dense ECM scaffold to aggregate and grow patient-derived tumor cells is more aligned with the term organoid in other publications. Nevertheless, these cultures maintain expression levels of ChgA, Syp, and SSTR2, which validate the neuronal and endocrine phenotype of these organoids over long-term culture. More recently, D’Agosto *et al.* (2023) cultured short-term PDTOs derived from a G2 lymph node metastatic siNET that was maintained in culture for several months. Upon characterization, the organoid cultures expressed similar siNET markers as the parent tumor sample via IHC, namely strong expression of CK8-18 and CDX2, and heterogeneous expression of CgA and SYP, and formed complex lumen-containing structures. Additionally, the proliferative index did not change significantly between organoid and tissue, maintaining the classification of G2. Compared to tumor organoids, there is a relative lack of research on the usage of 3D culture for siNET cell lines, including spheroids. Spheroids are cultured using non-adherent conditions and rely on microgravity to aggregate cells. Barazeghi *et al.* (2021) described in a recent publication that they successfully cultured non-adherent GOT1 spheroids to study cell proliferation when challenged with CPI-1205, a small molecule inhibitor of enhancer zeste homolog 2 (EZH2), and metformin, reinforcing recent data which suggest these tumors may be a result of epigenetic dysregulation. Most recently, Dayton e*t al.* (2023) developed a set of lower-grade small intestinal NET organoids from both primary and metastatic lesions that were positive for chromogranin A and had similar transcriptional profiles to their parent tissues. This maintenance of gene expression patterns between patient and model underlines the value of patient-derived organoid modeling for investigations into tumoral protein signaling and drug screening. One of the tested siNET organoid lines in this investigation showed no single nucleotide variants but demonstrated structural variants; this recapitulates what is known about the genetic basis of this disease in general (Elias *et al.* 2021). Overall, these 3D cultures provide a translational bridge between cell lines and *in vivo* models, and they often maintain an epithelial phenotype and provide a more conducive growth environment, given ECM support. In contrast with animal models and larger-scale *ex vivo* bioreactor modeling, organoids and spheroids maintain the high-throughput, reproducible nature required for screening assays. Combined with an apparent need for cell lines that are truly representative of siNETs, and the wide genetic heterogeneity among them, organoids provide a reproducible structure to analyze each small intestinal neuroendocrine tumor as a separate entity with a variety of *in vitro* assays. One potential area for future research and development in small intestinal 3D modeling includes culturing primary cells in ECM on a microfluidic chip to recapitulate the tumor immune microenvironment and cell-cell interactions more accurately within these tumors (Aref *et al.* 2018).

## Mouse models

*In vivo* modeling of tumor progression is one of the main hallmarks of preclinical investigation, which illuminates potential avenues toward innovation in therapeutics. There are three common routes for modeling tumors: patient-derived xenograft (PDX), cell line-derived xenograft (CDX), and genetically engineered mouse models (GEMMs), which produce sporadic or hereditary tumors over time. In recent literature, there have been successful attempts to implant both cell line- and patient-derived material into mice, which produce a tumor eligible for further experimentation (Table 3). However, due to the indolent and slow-growing nature of these tumors and their derivative cell lines, xenografting has only been sparsely successful in a select number of studies. As of this review, there are few successful accounts of a genetically engineered mouse model that produces sporadic or hereditary small intestinal lesions, although there have been successful GEMM developments in NETs of other origins, whether through selective silencing of genes or via oncogenic transformation (Forsythe *et al.* 2023).

**Table 3:** Current murine models of small intestinal neuroendocrine tumors.

Model	Type	Location	Notes	References
Transgenic, carrying insulin-promoted oncogenes	GEMM	Small intestine	Secreted secretin, proglucagon-related peptides, glucose-dependent insulinotropic polypeptide, among others. Large T-antigen immunoreactivity	Rindi *et al.* (1990)
GOT1 CDX/BALB/cABom-nu	Cell xenograft	Subcutaneous, back of the neck	IHC positive for major NET markers (CgA, Syp, NCAM)	Kölby *et al.* (2001)
Low-grade LM PDX/NOG	Tumor xenograft	Subcutaneous, hepatic orthotopic	Hepatic orthotopic was not successful, some cells from SQ were positive for NET markers	Hofving *et al.* (2021*b*)
CNDT2.5 PDX/NMRI-nude female	Cell xenograft	Subcutaneous, hind flank	Cells embedded in Matrigel, cells positive for Syp	Barazeghi *et al.* (2021)
RT2 transgenic mice, B6AF1 background	GEMM	Ileum	Tumors noted to have high expression of IGF2, which is a potential driver of tumorigenesis	Contractor *et al.* (2020)

In 1990, Rindi *et al.* (1990) reported the development of neuroendocrine tumors in the gastrointestinal tract of transgenic mice, some of which produced hormones including secretin and proglucagon-related peptides. This represented one of the earliest reports of a murine model for this cancer type. In 2001, Kölby *et al.* (2001) published one of the first successful transplantations of primary cells cultured from a liver metastasis of a small intestinal neuroendocrine tumor, which were subsequently established as the GOT1 line. The researchers transplanted tumor cells into male BALB/cABom-nu mice; over time, these tumors were resected and passaged over five generations. Small intestinal NET immunohistochemical markers were positive across all generations of tumor propagation. While studying the tumor microenvironment of siNETs, Hofving *et al.* (2012*b*) reported one successful attempt at subcutaneous grafting of cells from a low-grade liver metastasis of an siNET in a NOG mouse. The authors reported, however, that most attempts at both subcutaneous and hepatic orthotopic xenografting from dissociated cells were ultimately unsuccessful; only 1 of 36 subcutaneous grafting attempts was successful. Successive undigested tumor sample xenografting from the original patient was marginally successful, with a modest amount of live siNET cells after propagation, although tissue necrosis was noted. Via IHC, the metastatic siNET cell identity was verified with positive staining for synaptophysin. Most recently, in addition to GOT1 spheroid development, Barazeghi *et al.* (2021) documented successful xenotransplantation of CNDT2.5 cells to investigate EZH2 inhibitor therapy for siNETs. CNDT2.5 cells were embedded in Matrigel and subcutaneously injected into the hind flank of nude female mice. In all conditions, the xenografted tumor stained positively for synaptophysin and staining for CgA was not noted. Recently, Contractor *et al.* (2020) have reported the development of ileal neuroendocrine tumors, specifically in the B6AF1 genetic background, from the RT2 transgenic mouse model, which usually produces pancreatic neuroendocrine tumors.

## Other models of siNETs

Zebrafish are commonly used as a model of tumor angiogenesis and as a relatively reproducible animal model of neuroendocrine tumors, including those that arise from the small bowel. Gaudenzi *et al.* reported a successful xenotransplantation of primary culture from a liver metastasis of a low-grade (Ki67 < 2%) ileal NET in a teleost zebrafish with EGFP-labeled *fli1a* (Gaudenzi *et al.* 2017, Gaudenzi & Vitale 2019)*.* The EGFP-conjugated *fli1a* promoter provides a fluorescent marker for vascular development and tumor vasculature. Anti-fibroblast processing preceded the initial transplantation of suspended NET cells into the subperidermal space of the zebrafish embryos 24 h post fertilization. The authors measured a 33% tumor-induced angiogenesis ratio in fish with the PDX culture, and 67% of fish were positive for tumor cell migration. Notably, the authors reported an unsuccessful PDX attempt of a primary G2 ileal lesion. This specific class of model provides a bridge between organoid and mouse models to address tumor microenvironmental factors that may affect treatment efficacy on an organismal level, while being relatively higher-throughput compared to other animals.

## Limitations of current siNET models

Limitations of siNET models stem from the nature of the cancer from which they are derived; a slow-growing, indolent cancer generates indolent models. Additionally, the lack of single nucleotide genetic variants impacts animal model development. For example, the doubling time of GOT1 is 18 days, which presents a barrier for cell proliferation and growth assays and cell line xenografting in animal models. Another recurring issue in siNET model development is the inconsistent validation for available cell lines. Pfragner *et al.* reported the establishment of a continuous line, KRJ-I, from a small intestinal carcinoid tumor in 1996 (Pfragner *et al.* 1996). This cell line expressed both neuroendocrine markers and estrogen receptors, and these cells were morphologically representative of the original tumor; however, staining for many common NET antigens, including somatostatin receptors and VIP, was negative in the original culture. Recently, the validity of this line has been the subject of debate. In culture, KRJ-I cells grow in suspension, which is not consistent with other cell lines in this group. Additionally, these cells stained positive for lymphoblastoid markers, including CD45 and CD20. These issues call into question the utility of these cells for *in vitro* studies of siNETs (Hofving *et al.* 2018). However, this cell line is well cited in literature and mimics these tumors on a transcriptomic level (Alvarez *et al.* 2018, 2019). Hofving *et al.* classified L-STS and H-STS cell lines, which were originally identified as lymphatic and hepatic metastases from the primary lesion used to develop the P-STS line. The metastatic lines did not stain positively for many neuroendocrine markers or cytokeratins. These lines were also positive for lymphoblastoid markers and Epstein-Barr Virus DNA. Therefore, KRJ-I, L-STS, and H-STS are not representative models of siNETs (Hofving *et al.* 2018). Overall, examination and characterization of cell lines are important to determine which lines are suitable for experimentation, and researchers should demonstrate caution when using these cell lines.

Although CDX and PDX animal models have been recently developed using immunodeficient mice, this limitation attenuates the utility of these models for investigations into the tumor immune microenvironment *in vivo*. Only one genetically engineered mouse model resulted in the sporadic generation of small intestinal neuroendocrine tumors within immune-competent mice. In the study, Rindi *et al.* reported accounts of spontaneous generation of intestinal neuroendocrine tumors in RIP1Tag2/RIP2PyST1 mice in 1990 (Rindi *et al.* 1990); however, the precise location of these tumors was not noted, and this model has not been replicated since initial documentation. Although insulinomas, gastrinomas, glucagonomas, and non-functional pancreatic NETs have been grown in MEN1-mutant, RIP-Tag, and Glu-Tag murine models, there are no accounts of siNET generation (Pritchard 2022). Clinically, MEN1 patients often present with foregut and pancreatic tumors (Kamilaris & Stratakis 2019, Al-Salameh *et al.* 2021), but do not usually have midgut tumors in the jejunum and ileum. This may explain the relative lack of small intestinal lesions in this model specifically. Additionally, given the low mutational burden of these tumors, transgenic mouse models may not be feasible. However, unlike cell lines and organoid models, animal models would provide the most realistic representation of carcinoid syndrome, which is an important aspect of this disease (Vitale *et al.* 2023). This warrants further development of this class of model.

## Computational modeling

**Figure 3:**
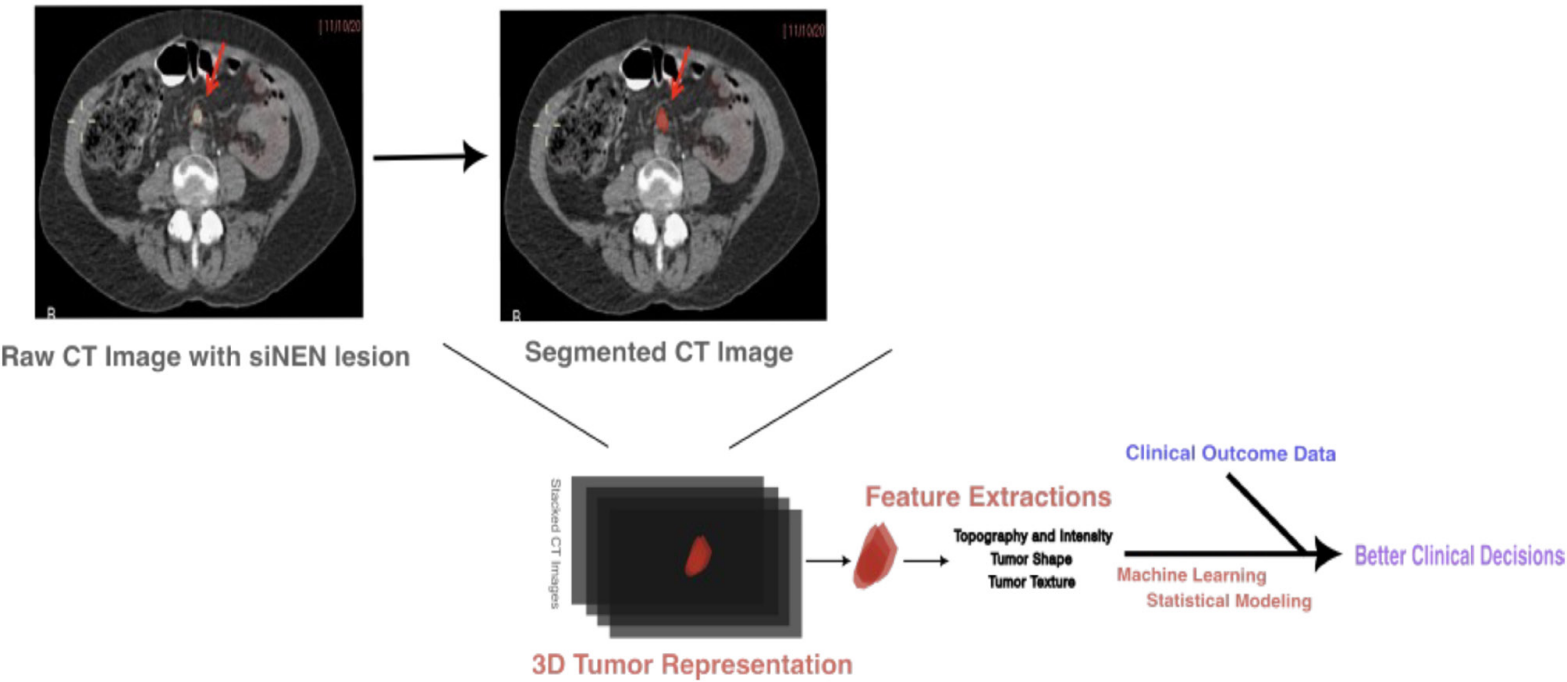
Model schematic of radiomic modeling of small intestinal neuroendocrine tumors using segmentation of computed tomography imaging.

Although *in vitro* and *in vivo* modeling provide necessary insights into tumor biology, computational modeling of small intestinal NET growth, grading, and therapy can help refine technology and improve patient outcomes. Radiomics is a powerful computational tool to model tumor growth for the prediction of prognosis (Fig. 3). Through a nomogram, high-depth data can be generated from radiologic scans to model tumor progression through computational algorithm training (Wu *et al.* 2017). Artificial intelligence and machine learning can be utilized as high-throughput tools to improve the algorithm over successive samples and provide deeper insights on prognostic factors (Lu *et al.* 2018).

Recently, this methodology has been applied to small intestinal neuroendocrine tumors. Blazevic *et al.* (2021) reported using radiomic analysis to predict small intestinal NET metastases in the mesentery. In this study, CT scans were digitally segmented for a region of interest and assessed by a trained clinician. Mesenteric mass and surrounding mesenteric regions were then quantified for various prognostic factors. A trained algorithm with various models interpreted the data to predict the development of adverse intestinal symptoms in patients, which the authors noted was likely due to the characteristic surrounding mesentery of the metastasis.

In addition, pharmacokinetics, the study of therapy uptake and metabolism, presents a clinically applicable mathematical model of siNET therapy and can be applied to increase the depth of *in vivo* studies. Given the current recommendations for peptide-receptor radionuclide therapy for siNET cases, modeling how this specific therapeutic works is a vital asset for investigating clinically relevant questions regarding absorption, toxicity, drug metabolism, half-life, and excretion. There are many different forms of PRRT, with different radiolabeling molecules, that have been subject to investigation as a therapeutic intervention for NETs.

Using ^212^Pb-SSTA-PBPK (physiology-based pharmacokinetic) mice as a model of alpha-PRRT, Zaid *et al.* (2021) demonstrated the value of combining a whole organism PBPK model for evaluating dosimetry of alpha-particle emitting therapy and toxicity in various SSTR-producing tissues with computational PK methods. Additionally, the authors analyzed renal toxicity of this treatment, which is vital in the transition of this therapy to the clinic, and found that including a kidney model could provide key insights into long-term renal toxicity as a result of PRRT. Jimenez-Franco *et al.* (2021) performed *in silico* modeling of ^177^Lu-DOTATATE therapy, which is currently FDA-approved for well-differentiated siNETs where SSTR expression is high. The researchers used a virtual patient PK model to investigate tumor control probability with respect to tumor perfusion and SSTR2 density; the results indicated a minimum tumor perfusion of 0.062 mL/g/min and receptor density of 55 nmol/L to establish a 99% tumor control probability. The authors noted that further computational analysis of dosimetry of PRRT is vital for optimization of this therapy for NETs.

## Future directions

As previously mentioned, small intestinal NETs are known to be genetically heterogeneous and show some clustering for limited aberrations at specific gene loci, including *CDKN1B* (Elias *et al.* 2021, Wedin *et al.* 2022). Current therapies for siNET, beyond surgical excision, are only moderately successful, depending on differential somatostatin receptor density, tumor cell differentiation and presence or absence of mutations among patients. Current models seek to improve our understanding of these factors and provide new avenues for treatment. For example, high-throughput organoid development protocols, ideally through patient biopsy, could be cultured, then challenged with a panel of therapeutics to determine efficacy on a per-patient basis. This precision oncology approach may be useful in the future when more drugs become FDA-approved for these malignancies. In the short term, organoid biobanking of siNETs expands patient samples for larger scale research projects.

Bioreactor tumor modeling has been used recently as a 3D *ex vivo* system for pancreatic NET tumor modeling and could theoretically be applied to NETs that arise from the small intestine (Herring *et al.* 2021). This technology uses a scaffold that holds cultured tumor cells in a matrix and continuously perfuses media through the scaffold. Imaging of these scaffolds is non-invasive and can be evaluated for growth via IR-783, a specific near-infrared fluorescent dye. Aref *et al.* (2018) published another microfluidic model, based on using patient-derived tumor spheroids on a chip to investigate immune checkpoint biology in siNETs. A mixture of tumor, immune, and stromal cells were embedded in collagen I and perfused with media. Although these initial studies are accounts of the developments in 3D modeling in the last 5 years, future innovation in this area could further close the gap that exists in siNET modeling, extend the utility of samples, and better recapitulate the tumor environment *in vivo*.

## Conclusion

As siNETs increase in incidence globally, there is a growing need in neuroendocrine tumor research, and specifically siNET research, for better models which provide representative insights, are cost-effective, and are higher throughput. There have been many attempts to increase the breadth and depth of siNET models (Table 4); many were unsuccessful or have failed tumor validation. Patient tissues from which these models are derived are rare, which limits development. However, organoid modeling is one of the best candidates to reflect the genetics and multicellular makeup of siNETs in larger-scale experiments when these tissues can be procured. Cell lines provide a lower-cost and broadly accessible alternative, albeit they are less representative of the original tumor. With advancements in tissue engineering, more intricate and sophisticated *ex vivo* modeling is also worthy of future research. Off the bench, *in silico* and computational models can provide translational insights with clinical applications. Given the intricacies of these specific tumors, models that are malleable yet best reflect the patient ultimately result in the most powerful data and preclinical innovation.

**Table 4:** Overview of currently available siNET models and their advantages and limitations in basic, translational, and clinical research.

Model	Examples	Intended use	Advantages	Limitations
**Cell lines**	GOT1 (met) (Kölby *et al.* 2001) P-STS (primary) (Pfragner *et al.* 2009) HC45 (met) (Stilling *et al.* 2007) CNDT2.5 (met) (Van Buren *et al.* 2007) SS-C (functional) (Galli *et al.* 2006) STC-1 (mouse) (Rindi *et al.* 1990)	*In vitro* experimentation where fast growth is required, cell-based assays	Highest-throughput, highly reproducible and easy to generate more, easily manipulated for assays, cost-effective	Least representative, some lines harbor uncharacteristic mutations or lack biomarkers, immortalization procedures necessary to extend growth timespan
**Spheroid**	GOT1-derived spheroids (Barazeghi *et al.* 2021)	*In vitro* experimentation, drug screening, functional assays	Maintain multicellular aggregates, providing more representative drug responses than cell lines	Lack of tissue architecture, finite growth timeline, size limitations
**Organoid**	Short-term siNET PDOs (Ear *et al.* 2019) G2 LN Met PDOs (D’Agosto *et al.* 2023) Low-grade siNET Primary and Met PDOs (Dayton *et al.* 2023)	*Ex vivo* experimentation, drug screening, long-term culture	Maintain varied tumor cell populations *ex vivo*, representative drug response, culture is representative of an individual patient, high throughput	Limited growth timespan, require rare tumor tissue, more time- and cost-intensive
**Zebrafish**	PDX EGFP-*fli1a* telost (Gaudenzi *et al.* 2017)	*In vivo* experimentation related to tumor angiogenesis, migration, and metastasis	Specialized model for studies of vasculature and migration, high xenograft establishment rate compared to other animal models	Specialized equipment requirements, limited scope of use, requires rare patient tissue
**Mouse**	Transgenic, insulin-promoted oncogenes (Rindi *et al.* 1990) GOT1 CDX/BALB/cABom-nu (Kölby *et al.* 2001) Low Grade LM PDX/NOG (Hofving *et al.* 2021*b*) CNDT2.5 PDX/NMRI-nude female (Barazeghi *et al.* 2021) RT2 transgenic (Contractor *et al.* 2020)	*In vivo* experimentation, preclinical modeling, testing therapy candidates	Most representative preclinical model, standard for translational drug efficacy studies	Most cost-intensive, lowest throughput, ethical and institutional regulations with animal use in research, lowest rate of model establishment
**Clinical**	siNET CT radiomics (Blazevic *et al.* 2021) siNET therapy pharmacokinetics (Zaid *et al.* 2021, Jimenez-Franco *et al.* 2021)	Computational modeling for clinical decision-making, drug uptake studies, and macroscopic tumor analysis	Can accommodate large, already available datasets and improve patient outcomes	Limited use for basic or translational research, requires powerful computational resources

## Declaration of interest

The authors declare that there is no conflict of interest that could be perceived as prejudicing the impartiality of this review.

## Funding

Funding is provided by the National Institutes of Healthhttp://dx.doi.org/10.13039/100000002 ZIA BC 011899.

## Consent for publication

All authors have read and approved the final manuscript.

## Availability of data and materials

The datasets used and/or analyzed during the current study are available from the corresponding author on reasonable request.

## Author contribution statement

ASG – conceptualization, manuscript writing, figure preparation, editing. FSD – manuscript preparation, expert opinion, editing. MJP – manuscript preparation, expert opinion, editing. SSM – conceptualization, oversight, expert opinion, manuscript editing.
